# Molecular genetics of Parkinson’s disease: Contributions and global trends

**DOI:** 10.1038/s10038-022-01058-5

**Published:** 2022-07-11

**Authors:** Manabu Funayama, Kenya Nishioka, Yuanzhe Li, Nobutaka Hattori

**Affiliations:** 1 Research Institute of Disease of Old Age, Graduate School of Medicine, Juntendo University, 2-1-1 Hongo, Bunkyo-ku, Tokyo, 113-8421 Japan; 2 Department of Neurology, Juntendo University School of Medicine, 2-1-1 Hongo, Bunkyo-ku, Tokyo, 113-8421 Japan; 3 Neurodegenerative Disorders Collaborative Laboratory, RIKEN Center for Brain Science, 2-1 Hirosawa, Wako, Saitama, 351-0106 Japan

**Keywords:** Parkinson's disease, Genome-wide association studies, Medical genomics

## Abstract

Parkinson’s disease (PD) is a neurodegenerative disorder primarily characterized by motor dysfunction. Aging is the greatest risk factor for developing PD. Recent molecular genetic studies have revealed that genetic factors, in addition to aging and environmental factors, play an important role in the development of the disorder. Studies of familial PD have identified approximately 20 different causative genes. *PRKN* is the most frequently detected causative gene in Japan. The *PRKN* gene is located at a common fragile site, and both copy number variants as well as single nucleotide variants are frequently detected. The location and variety of variant types makes an accurate genetic diagnosis difficult with conventional genetic testing. In sporadic PD, genome-wide association studies have revealed more than 200 genes that are potential drivers for the development of PD. Many of these studies have been conducted in Caucasian populations alone, which has limited the identification of all genetic risk factors for sporadic PD, particularly as genetic backgrounds vary widely by race. The Global Parkinson’s Genetics Program is a global undertaking meant to address the issue of regional differences in genetic studies of PD.

## Introduction

**Fig. 1:**
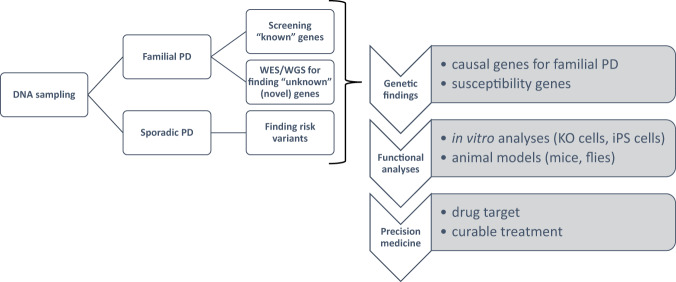
Strategies to identify genetic factors in PD and applications of the findings. The search for genes associated with PD relies on the molecular genetic characteristics of PD patients and the classification of samples into familial PD and sporadic PD groups. After PD-associated genes are discovered, cellular and animal PD models are used to reveal pathophysiological functions and for precision medicine applications

Parkinson’s disease (PD) is a progressive neurodegenerative disease whose main symptoms are motor dysfunctions, such as tremor, rigidity, bradykinesia, and postural instability. Selective degeneration of dopaminergic neurons in the substantia nigra of the midbrain underlies these symptoms. PD patients often have comorbid non-motor symptoms that include autonomic dysfunctions, such as constipation and orthostatic hypotension, as well as psychiatric symptoms, such as anxiety and depression. Thus, PD is not a disease of the central nervous system alone, but rather a systemic disease. Because the prevalence of PD increases with age, it is clear that aging is a risk factor for its development. Environmental factors are also thought to be important, as exposure to pesticides and other chemicals can cause PD symptoms. Advances in genetic research methods have provided insights into how genetic backgrounds contribute to the development of PD. Accordingly, PD is a complex genetic disease that is caused by a combination of aging, environmental factors, and genetic factors. There are two major ways to identify genetic factors in PD (Fig. 1). One is to investigate rare Mendelian forms of PD and identify the causative genes. The other is to identify risk variants from genetic statistical analyses of large groups of subjects. While there are advantages and disadvantages in each approach, both are necessary to provide a more complete picture of this disease. Genetic studies have identified over 200 PD-related genes [1]. However, regional differences, associated with variations in ethno-social factors, have complicated molecular genetic studies of PD. Here, we discuss the current status and challenges of molecular genetics research on PD.

## Molecular genetics of Mendelian forms of PD

It has been a quarter of a century since the *alpha-synuclein* gene (*SNCA*) was reported as the causative gene for the autosomal dominant form of PD [2]. In the early days of Mendelian PD research, the prevailing approach used to discover disease-related genes and variants included identifying loci from positional cloning of large families, using artificial chromosome libraries, such as yeast artificial chromosomes (YACs) and bacterial artificial chromosomes (BACs) to detect the genes and identify patient-specific variants by sequence analysis. This approach led to the discovery of *SNCA* and *PRKN* [2, 3]. With the completion of the Human Genome Project and the advent of next-generation sequencers, the throughput has been dramatically improved, and the resulting gene maps and sequences have become more readily available [4]. Together, these molecular genetic Mendelian PD studies have identified 24 genes or loci that have been registered in the Online Mendelian Inheritance in Man (OMIM) database as being involved in the development of PD (Table 1).

**Table 1:** PD-related genes registered in OMIM

Loci	Chromosomal position	Gene
*PARK1/4*	4q21.3-q22	*SNCA*
*PARK2*	6q25.2-27	*PRKN*
*PARK3*	2p13	*unknown*
*PARK5*	4p13	*UCH-L1*
*PARK6*	1p36.12	*PINK1*
*PARK7*	1p36.23	*DJ-1*
*PARK8*	12q12	*LRRK2*
*PARK9*	1p36	*ATP13A2*
*PARK10*	1p32	*unknown*
*PARK11*	2q36-q37	*GIGYF2*
*PARK12*	Xq21-q25	*unknown*
*PARK13*	2p13.1	*HTRA2*
*PARK14*	22q13.1	*PLA2G6*
*PARK15*	22q11.2-qter	*FBXO7*
*PARK16*	1q32	*unknown*
*PARK17*	16q12	*VPS35*
*PARK18*	3q27-qter	*EIF4G1*
*PARK19*	1p31.3	*DNAJC6*
*PARK20*	21q22.11	*SYNJ1*
*PARK21*	3q22.1	*DNAJC13*
*PARK22*	7p11.2	*CHCHD2*
*PARK23*	15q22.2	*VPS13C*
*PARK24*	10q22.1	*PSAP*

### PRKN

**Fig. 2:**
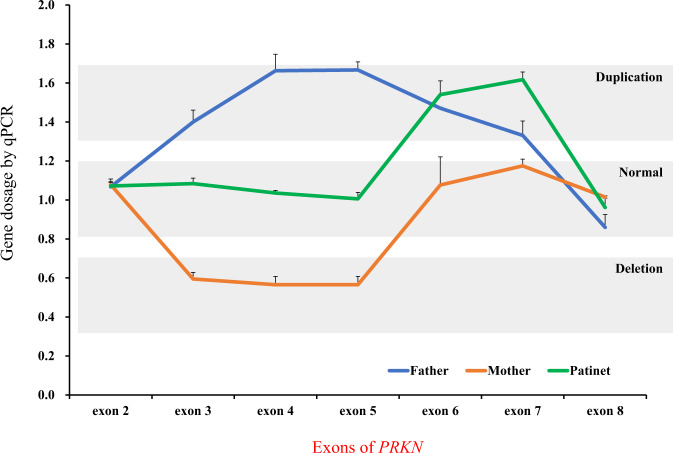
Pseudo-heterozygotes in the *PRKN* gene. Copy number variations tested by qPCR showed that the PD patient (green) had a heterozygous duplication in exons 6–7. The PD patient inherited a duplication of exons 3–7 from the father (blue) and a deletion of exons 3–5 from the mother (orange), resulting in compound heterozygous variants in the *PRKN* gene

The most frequent causative gene of PD in Japan is the *PRKN* gene, which is the causative gene of autosomal recessive juvenile PD, as reported by Kitada et al. in 1998 [3]. We recently published the results of a study of the *PRKN* gene in over 2000 cases, and identified biallelic variants in *PRKN* that are observed in 8.1% (98/1204) of familial PD and 5.8% (65/1118) of sporadic PD cases [5]. It is important to note that this population included many patients who were clinically diagnosed as likely to have *PRKN* variants and were actively selected for genetic analysis. Therefore, the sampling bias is likely to have been strong, and the frequency of *PRKN* variants is estimated to be a little lower than that observed in the study. We also found PD patients with a putative pathogenic monoallelic rare variant in the *PRKN* gene, with a frequency of 2.5% (57/2322). A comparison of clinical symptoms between the biallelic and monoallelic variants showed that the age at onset was 29.6 ± 9.8 years for the biallelic variants and 45.2 ± 15.9 years for the monoallelic variant, which indicates an earlier age of onset for the biallelic variants [5]. Additionally, more cases of Hoehn and Yahr stage IV or V were observed in the biallelic variants in patients who had the disease for at least 15 years [5]. The *PRKN* gene is the causative gene of autosomal recessive juvenile PD, and therefore, a person with a monoallelic variant is presumed to be an unaffected carrier. However, the frequency of the *PRKN* monoallelic variant is slightly higher in Japanese PD patients than the *Leucine rich-repeat kinase 2* (*LRRK2*) variant, a cause of autosomal dominant PD (discussed further below). Thus, the *PRKN* monoallelic variant may have some influence on the development of PD. Because the *PRKN* gene is located at a common fragile site, it is prone to site-specific gaps and breaks due to chromosomal fragility [6]. Indeed, the *PRKN* gene is a cluster of deletions and duplications in exons 2 through 7. Quantitative PCR (qPCR) and Multiplex Ligation-dependent Probe Amplification (MLPA) are simple and widely used tests for copy number variations such as deletions and duplications. However, these methods only target a part of a gene, and the results therefore need to be interpreted carefully. For example, we reported a pseudo-heterozygous variant in a PD patient whose father had a duplication in exons 3–7 and whose mother had a deletion in exons 3–5. The patient inherited the mutant allele from both parents and developed juvenile PD, but was incorrectly identified as having a heterozygous duplication in exons 6–7 due to the gene dosages of the parental mutant alleles canceling each other out in qPCR testing (Fig. 2) [7]. Thus, genetic testing of only the patient may miss the biallelic variants, and therefore, it is advisable to test the patient as well as close relatives, including those who have not yet developed the disease. However, some variants, such as variants in the promoter region and small inversions involving only a partial region of the *PRKN* gene, may be difficult to identify even with trio analysis. When small inversions are present, the abnormality cannot be detected by sequence analysis using the Sanger method or by copy number variation analysis using the qPCR or MLPA methods. Therefore, even if one genetic test is negative, diligent testing should be continued if there is clinical suspicion of gene mutation [8]. The long-read sequencing method is a useful genetic analysis method that should help identify the underlying genetic variants.

**Fig. 3:**
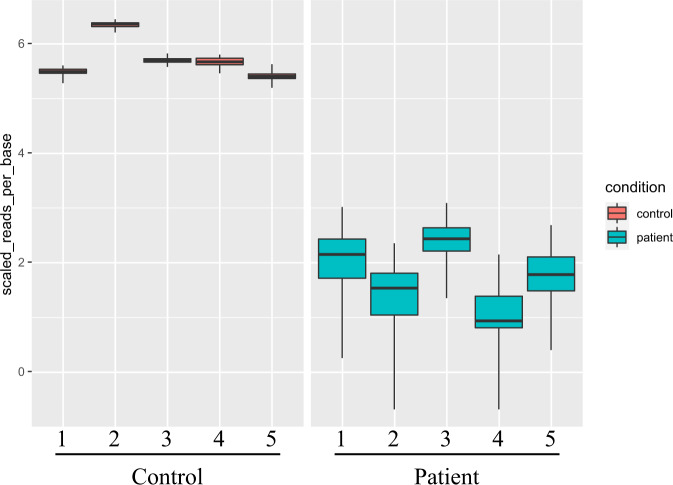
RNAseq analysis of autosomal recessive juvenile parkinsonism (ARJP) patients with *PRKN* single heterozygous variants. RNAseq analysis of five ARJP patients (right half) and five controls (left half) shows one example of a gene whose expression is prominently and significantly downregulated only in ARJP patients

For a more complete analysis, it is necessary to consider genes other than *PRKN* that are involved in the pathogenesis of the disease. We have conducted RNAseq analysis of patients with both a monoallelic variant in the *PRKN* gene and the juvenile PD phenotype, and found patient-specific gene expression changes (Fig. 3). Although this downregulated gene has a different chromosomal location from the *PRKN* gene, it has been reported to be expressed in the nervous system and may be functionally related to *PRKN*. Thus, for autosomal recessive diseases with monoallelic variants in genes such as *PRKN*, other loci should be considered. These strategies include (1) seeking deletions, duplications, and often-missed biallelic variants, such as those in transcriptional regulatory regions, (2) seeking biallelic or polygenic variants, (3) investigating patient susceptibility to the disease, and (4) considering other unrelated causes. Therefore, it is necessary to make careful assessments based on family history and clinical considerations in the diagnosis of PD.

### PTEN-induced kinase 1 (PINK1)

The second most frequent gene after *PRKN* as a causative gene for autosomal recessive PD is *PINK1*. *PINK1* is a PD gene discovered in 2004 from a genetic analysis of families in Sicily, Italy [9]. Subsequent follow-up studies revealed many PD-related variants. PINK1 is a mitochondrial kinase. Single nucleotide variants (SNVs) in *PINK1* are more common than copy number variants (CNVs) in *PRKN*. We performed genetic analysis of *PINK1* in 1700 PD patients and identified *PINK1* variants in 1.8% of young-onset familial PD and 0.009% of young-onset sporadic PD [10]. PINK1 and parkin are known to cooperate and are involved in mitochondrial quality control [11]. We found a total of four PD patients with digenic *PRKN*-*PINK1* variants [10, 12]. These digenic variants seem to have younger age of onset than biallelic variants in *PINK1* or *PRKN* [12]. However, the small number of cases makes it unclear whether these differences are significant.

### Leucine rich-repeat kinase 2 (LRRK2)

The most frequent PD causative gene in Europe and the United States is *LRRK2*, which was first reported in 2004 as the causative gene of the autosomal forms of PD [13, 14]. Originally, the *LRRK2* gene was mapped to chromosome 12 in a study of Japanese families with an autosomal form of PD [15]. The causative locus, *PARK8*, was mapped to chromosome 12 and linked to *LRRK2*. Although this gene is historically associated with Japan, the frequency of the *LRRK2* gene in PD in Japan is very low compared with Caucasians [16]. Conversely, the *LRRK2* gene p.G2019S variant is highly prevalent in Europe, the United States and Arab countries, including those in North Africa [17]. We analyzed approximately 1400 PD patients and estimated that the frequency of *LRRK2* variants in Japan is 1.7% in familial PD, 0.3% in sporadic PD, and 1.0% in all PD patients [16]. Most PD patients with *LRRK2* variants had families in which the mode of inheritance was thought to be autosomal dominant. However, some PD patients appeared to have sporadic PD with no known cases in the family, indicating incomplete penetrance. Clinical features such as tremors (78.3%), postural instability (73.9%), gait with small steps (55.6%), constipation (40.0%) and olfactory disturbances (41.7%) were also frequently observed, and there was no significant difference in clinical symptoms among pathogenic variants [16].

### Other genes in familial PD

In 2011, *Vacuolar protein sorter-35* (*VPS35*) was reported as the first causative gene for autosomal dominant PD based on whole-exome analysis using next-generation sequencing [18]. Since the report of *VPS35*, one or two genes a year have been reported as causative PD genes by studies using the same methodology. In a report from Japan, *coiled-coil-helix-coiled-coil-helix domain containing 2* (*CHCHD2*) and *prosaposin* (*PSAP*) were reported as novel genes for familial PD, though they have a very low frequency in Mendelian PD [19, 20]. *CHCHD2* is the first PD-causing gene involved in the mitochondrial respiratory chain complex, and *PSAP* is also involved in a lysosomal disease, similar to *glucocerebrosidase* (*GBA1*) [19, 20]. Despite their very low frequencies, these genes are important for understanding the pathogenesis of PD and as potential therapeutic targets. In particular, age-dependent decline in motor function and loss of nigrostriatal dopaminergic neurons have been observed in mouse models of *PSAP*, suggesting that *PSAP* may be useful as an animal model of PD [20, 21]. Other familial PD-causing genes include *DJ-1*, *ATP13A2*, *GIGYF2*, *HTRA2*, *PLA2G6*, *FBXO7*, *EIF4G1*, *DNAJC6*, *SYNJ1*, and *DNAJC13*, all of which are infrequent in Japanese PD. However, the functions of these gene products, including intracellular trafficking, oxidative stress, mitochondria, phospholipid membranes and ubiquitin-proteasome system, are all predicted to be involved in the pathogenesis of PD [22–31].

### Difficulties in discovering new PD-causing genes

One of the problems with Mendelian PD research is the lack of research targets. It has been reported that the percentage of Mendelian-inherited diseases for which the cause could be identified by whole-exome analysis is about 20% [32]. Because PD is a complex genetic disease with a late onset, the rate of cause-identification is expected to be even lower. One approach to overcome this limitation is to collect as many subjects as possible in the same family with Mendelian PD. In the case of manifest PD, collecting more genetic samples over multiple generations yields a higher probability of identifying the cause of the disease, but in many cases, the parents or grandparents with PD had died by the time the proband developed the disease. For example, in the *PARK8* family mentioned above, the causative gene locus was successfully identified after more than 20 years of intra-family research and DNA sampling, including from those who did not develop the disease [33–35]. Even though genetic analysis technology has evolved, and high-throughput techniques have improved dramatically, new discoveries will likely remain difficult to make. For example, when whole-exome sequencing is performed using small PD families, hundreds of candidate causative variants will be identified; however, with few exceptions, there is only one causal variant per family, regardless of its size. Investigators must therefore implement various techniques to narrow down the number of candidate variants from hundreds to one. In familial PD studies, it is expected that for at least 80% of the families, a single candidate variant and the causative gene will remain unidentified.

## PD risk genes and variants

### Genome-wide association studies (GWAS)

GWAS have been conducted to identify genetic factors that contribute to the pathogenesis of sporadic PD. To identify these factors, GWAS and meta-analysis on GWAS (Meta-GWAS) have been employed. In GWAS, microarray technology is used to genotype relatively frequent single nucleotide polymorphisms (SNPs). SNPs with large differences in frequency between the patient and control groups can be identified. Genes located near hit-SNPs, which are significantly more or less frequent in the patient group, are presumed to be more likely to influence the development of sporadic PD.

As a result of multiple GWAS, several important genes have been identified as being risk factors for PD. Among them, *SNCA* and *LRRK2* were found to be causative genes for the Mendelian forms of PD, but GWAS revealed that they are also involved in the development of sporadic PD [36, 37]. Two GWAS studies in Europeans and Japanese populations have shown that *LRRK2* is a common risk factor for PD regardless of race. However, Europeans share a unique variant, p.G2019S, while p.R1628P and p.G2385R are variants unique to Asians [38, 39]. According to the GWAS catalog, about 200 genes related to PD have been reported so far [1]. Furthermore, a recent meta-analysis of GWAS identified 90 independent variants in 78 genomic regions associated with PD; however, how these variants affect pathogenesis remains largely unknown [40]. Therefore, it is likely that more genes will be reported to be involved in PD pathogenesis in future Meta-GWAS. Clarifying how these many genes may be related to or affect PD onset as well as other clinical manifestations may require new approaches that combine artificial intelligence, deep learning, gene expression data, metabolomics, and other technologies. The findings from these combined approaches are expected to contribute to the prediction of PD onset and prognosis as well as to the implementation of targeted medicine strategies and therapeutics.

### GBA1

*GBA1* is the causative gene for Gaucher disease (GD), a lysosomal disorder [41]. GD is a recessively inherited disease caused by variants in the *GBA1* gene. Notably, in GD families, many GD-naive *GBA1* variant carriers have family members who develop PD [42, 43]. This suggests that a monoallelic *GBA1* variant may be a risk factor for developing PD, and in fact, *GBA1* was found to be a common and frequent risk factor for the development of PD in different populations, including Japan [44, 45]. Variants of *GBA1* known to be associated with PD development include p.E326K, p.T369M, p.N370S and p.L444P, which decrease the activity of glucocerebrosidase, the enzyme encoded by *GBA1*, and consequently reduce the ability to degrade alpha-synuclein in lysosomes [46]. Variants of *GBA1* have different frequencies in different races. For example, p.E326K has a frequency of 1–5% in the general European population, but is very rare in Asia.

### Polygenic risk score (PRS)

PD is thought to be caused by a complex interaction of multiple genetic risk factors with environmental factors and aging. The identification of novel risk genes and variants for PD by GWAS is a promising approach for discovering a small number of common causative genes or risk variants. The PRS is calculated for each individual based on the weighted sum of the risk variants, assuming that the patient has many risk variants. PRS has been shown to correlate with the actual risk of developing the disease, and by examining the distribution of scores within a population, it is possible to identify individuals at particularly high risk for the disease. Foo et al. identified 11 PD-risk SNVs in a meta-GWAS of Asian samples [47]. Two of these (*SV2C* and *WBSCR17*) were novel PD risk genes. The PRS was calculated using these SNVs, and it was shown that the age of onset decreased with each additional copy of the risk allele [47]. Similar PRS calculations using the previously reported 90 risk variants in a European sample showed no significant difference in risk prediction in spite of the 8-fold greater number of SNVs [47]. Thus, these findings suggest that the PRS calculation based on GWAS for each race is significant for disease prediction, and that the weights of individual risk SNVs differ by race.

### Efforts to address regional and racial differences in PD

Genetic regional and racial differences in PD are observed for many genes. For example, the *MAPT* gene, encoding the microtubule-associated protein tau, was detected as a risk factor for PD in a GWAS of a Caucasian population, but not in a GWAS of a Japanese population [36, 37]. However, several Japanese families have been affected by variants in *MAPT* [48, 49]. Most molecular genetic studies of disease have been conducted primarily in Caucasians for various geopolitical, cultural, linguistic, and economic reasons [50]. However, as the examples of *GBA1* and *MAPT* show, the lack of molecular genetic studies in multiracial populations is an important issue that must be resolved in future PD research.

The Global Parkinson’s Genetics Program (GP2) is a worldwide international consortium established under the auspices of Aligning Science Across Parkinson’s (ASAP) to actively promote research in regions where genomic research has not been actively conducted before and where English is not the native language [51]. For this reason, the GP2 web page (https://gp2.org) is available in Arabic, Chinese, French, German, Japanese and Spanish, in addition to English. GP2 has hubs in various locations, such as the East Asian Parkinson Disease Genomics Consortium (EAPDGC) in East Asia, which includes Japan, the Genomics Consortium Africa (IPDGC Africa) in Africa, and the Latin American Research Consortium on the Genetics of PD (LARGE-PD) [52–54]. Each hub collects thousands of patient and control samples to determine racial genetic background, polygenic risk scores, and other metrics. GP2 makes its data and analysis scripts available to the public and runs educational programs on how to use them to perform various analyses in the cloud. Through such open science, GP2 is expanding its reach beyond Caucasians to include both patients who have not previously been included in studies and researchers who have not previously had the opportunity to conduct large-scale genomic analysis studies.

## Conclusion

In this review, we described the current status of molecular genetics research on PD, its challenges, and the efforts to address them. Studying the molecular genetics of PD is a crucial first step in understanding the disease, because it advances our knowledge of the relationship between phenotype and genotype. Whether by revealing the full etiopathogenesis of the disease, uncovering novel pathogenetic mechanisms, or leading to other exciting findings, future molecular genetic studies are likely to foster major advances in our understanding of this debilitating neurodegenerative disorder in the near future.
